# The impact of the duration of the integrated disease management program on COPD-related outcomes

**DOI:** 10.1186/s40001-023-01136-0

**Published:** 2023-05-23

**Authors:** Ching-Hsiung Lin, Yi-Rong Li, Bing-Yen Wang, Sheng-Hao Lin, Kuo-Yang Huang, Cheng-Hsiung Chen, Chew-Teng Kor

**Affiliations:** 1 Division of Chest Medicine, Department of Internal Medicine, Changhua Christian Hospital, 135 Nanhsiao Street, Changhua, 50006 Taiwan; 2 Institute of Genomics and Bioinformatics, National Chung Hsing University, Taichung, Taiwan; 3 National Chung Hsing University, Taichung, Taiwan; 4 Department of Recreation and Holistic Wellness, MingDao University, Changhua, Taiwan; 5 Thoracic Medicine Research Center, Changhua Christian Hospital, Changhua, 500 Taiwan; 6 Department of Surgery, Division of Thoracic Surgery, Changhua Christian Hospital, Changhua, 500 Taiwan; 7 Big Data Center, Changhua Christian Hospital, Changhua, 500 Taiwan; 8 Graduate Institute of Statistics and Information Science, National Changhua University of Education, Changhua, 500 Taiwan

**Keywords:** COPD, Integrated care model, Intervention duration, MCID improvement for CAT, Exacerbation

## Abstract

**Background:**

The aim of this study is to assess the impact of the duration of the integrated disease management (IDM) program on COPD-related outcomes in real-world setting.

**Methods:**

A retrospective cohort study among 3771 patients with COPD who had regularly completed 4 visits of IDM program within 1 year between April 1, 2017 and December 31, 2018. CAT score as the primary outcome used to investigate the association between IDM intervention duration and improvement in CAT score. Change in CAT score from baseline to each follow-up visit determined by using least-squares means (LSMeans) approach. The cut-off value of IDM duration for improving the CAT score was determined by the Youden index. Logistic regression was used to analyze the relationship between IDM intervention duration and MCID (the minimal clinically important difference) improvement in CAT score and the factor associated CAT improvement. Risks of COPD exacerbation events (COPD-related ED visit and COPD-related hospitalization) were estimated by using the cumulative incidence curve and Cox proportional hazards models.

**Result:**

Among 3771 enrolled COPD patients, the majority of the study cohort were males (91.51%) and 42.7% of patients had CAT score of ≥ 10 at baseline. The mean of age was 71.47 years and the mean CAT at baseline were 10.49. The mean change from baseline in CAT score was − 0.87, − 1.19, − 1.23 and − 1.40 at 3-, 6-, 9- and 12 month follow-up (p < 0.0001 for all visits), respectively. Statistically significantly lower likelihood of achieving MCID improvement in CAT were observed at 3- and 6 month compared to 9 month (at 3 month: OR: 0.720, 95% CI 0.655–0.791; at 6 month: OR: 0.905, 95% CI 0.825–0.922). And only a modest increase likelihood of achieving MCID improvement in CAT at 12 month (OR: 1.097, 95% CI 1.001–1.201) compared with 9-month follow-up. In logistic regression on the entire cohort, CAT MCID improvement was most associated with baseline CAT scores ≥ 10, followed by frequent exacerbation in previous year (> 2 episodes/year), wheezing, and GOLD B or D at baseline. In baseline CAT ≥ 10 group, patients were more likely to achieve CAT MCID improvement and had greater decreases from baseline in CAT score observed at 3-, 6-, 9-, and 12 month compared with baseline CAT score < 10 group (all p < 0.0001). Moreover, in CAT ≥ 10 groups, patients who achieved CAT MCID improvement had lower risk of subsequent COPD exacerbation events (COPD-related ED visit: aHR: 1.196, 95% CI 0.985–1.453, p = 0.0713; COPD-related hospitalization: aHR: 1.529, 95% CI 1.215–1.924, p = 0.0003) when compared to those without.

**Conclusion:**

This is the first real-world study indicating the association between COPD IDM intervention duration and COPD-related outcomes. From 3 to 12 month follow-up results showed that continued improvement over time in COPD-specific health status, particularly in patients with baseline CAT score of ≥ 10. Furthermore, a reduction of the risk of subsequent COPD exacerbations were observed in patients with CAT MCID improvement.

**Supplementary Information:**

The online version contains supplementary material available at 10.1186/s40001-023-01136-0.

## Introduction

Chronic obstructive pulmonary disease (COPD) is a chronic, multi-factorial systemic disease worldwide with significant morbidity, which incurs heavy utilization of healthcare resources [1]. As a nonreversible disease, primary treatment goals of COPD aim to relieve symptoms and limit exacerbations while maximising functional ability and wellbeing [2]. However, fluctuating symptoms, various disabilities, and varying levels of well-being often complicates medical care of COPD [3]. In the last decade, the integrated care model (IDM), a multi-disciplinary and multi-component programme, has been proposed as an optimal strategy to address the challenges in COPD management [3–5].

A recent meta-analysis of 52 randomized trials demonstrated that IDM programme with a follow-up period at least 3 months hold the promise to improve disease-specific quality of life (QoL) and exercise capacity and demonstrated reduction in hospital admissions and hospital days per person [3]. In real-world evidence, a nationwide COPD IDM program in Taiwan exerted a positive net effect on reducing the likelihood of COPD exacerbation, such as COPD-related ED visits and hospitalizations [6]. An improved health status was also found in patients with COPD who received care according to the IDM program, namely COPDnet [7]. While RCTs and real-world studies have shown the efficacy and effectiveness of IDM programs on COPD management, the appropriate intervention duration is still unknown.

Recently, appropriate intervention duration of IDM program have been recommended to be a measure for assessing the cost-effectiveness on IDM program [8]. However, to the best of our knowledge, there are no study regarding the impact of duration of IDM program on COPD-related outcomes. Previous meta-analysis study revealed that the beneficial effects of COPD IDM program on health-related quality of life (SGRQ: St. George’s Respiratory Questionnaire) are statistically significant in the short term (up to 6 months) and in the medium term (6 to 15 months). IDM probably results in a reduction in emergency department (ED) visits and a fewer hospital days per person admitted with median follow-up 12 months [3]. However, there is no conclusion about the more effective intervention duration for COPD IDM program. In the Netherlands, a 2 year cluster RCT in 40 general practices found that IDM program for patients with COPD in primary care increased costs without improvement in health outcomes [9]. Consequently, the inappropriate intervention duration of IDM may not improve quality of care for patients with COPD and increase the financial burden on the health care system.

The aim of this study is to evaluate the impact of the intervention duration of the COPD IDM program on COPD-related outcomes in real-world settings. We first investigate influence of intervention duration on the change in COPD-specific QoL using CAT score as an indicator. In addition, we further assess the association between intervention duration and CAT MCID improvement. We further analyzed the factors associated with CAT MCID improvement, and risk of subsequent COPD exacerbation between patients with or without COPD-specific QoL.

## Material and methods

### The protocol of COPD integrated care (IDM) program

A COPD IDM p program, Taiwan COPD P4P, was investigated in this study. The protocol included patient criteria, component of disease management, and multidisciplinary team have been described previously [6]. In brief, this program was described as follows: patients diagnosed with COPD (ICD10 codes: J41-J44) and their diagnosis was confirmed using a spirometer (postbronchodilator FEV1/FVC < 70%) within the 90 days before program enrollment. Patients who met the inclusion criteria received comprehensive pharmacologic and nonpharmacologic treatment from a multi-disciplinary team consisting of pulmonary specialists, otorhinolaryngologists, pediatric specialists, family medicine specialists, respiratory therapists, pharmacists, and case managers. And diseases management based on Taiwan’s COPD guidelines [10]. The nonpharmacological intervention included pulmonary rehabilitation, smoking cessation, patient and family education, as well as integrated disease-specific information and health care resource integration. The enrolled patients had their COPD treatment managed and medications adjusted by physicians. Additionally, case managers provided each patient with a personalized education program that included all essential disease-specific information. The respiratory therapist provided instructions for pulmonary rehabilitation course. An enrollee of COPD IDM program is advised to visit the physicians once every 3 months. Implemented structured care is clearly defined in initial enrollment visit (baseline), continuing care visits (visit 1: at 3 months; visit 2: at 6 months; visit 3: at 9 months), and first annual evaluation visit (visit 4: at 12 months), respectively. IDM program participants continued to participate in subsequent rounds (visit 5, and so on) as required. If the patients who had not come for follow-up over 3 months, refuses to follow doctor’s orders, then the case will be closed.

### Data sources

Data used in this study extracted from Taiwan COPD P4P registry dataset and Taiwan National Health Insurance (NHI) claim datasets. Among the data collected from the Taiwan COPD P4P registry dataset were BMI (body mass index), COPD Assessment Test (CAT) scores, the mMRC (Modified Medical Research Council) Dyspnea Scale, smoking status, post-bronchodilator spirometry data, and GOLD risk group in COPD IDM program. A NHI claim dataset was used to collect demographics, outpatient and inpatient claims, diagnostic codes on the basis of the International Classification of Diseases Ninth Revision Clinical Modification (ICD-10-CM). The study was conducted according to the guidelines of the Declaration of Helsinki. Changhua Christian Hospital's Institutional Review Board approved the present study (Approval Number: 200904). An informed consent was waived since the research was retrospective in nature. All methods were carried out in accordance with relevant guidelines and regulations by ethics committee. Data from the database was de-identified. Taiwan’s Computer-Processed Personal Data Protection Law and privacy regulations were followed by researchers.

### Study design and participants

**Fig. 1:**
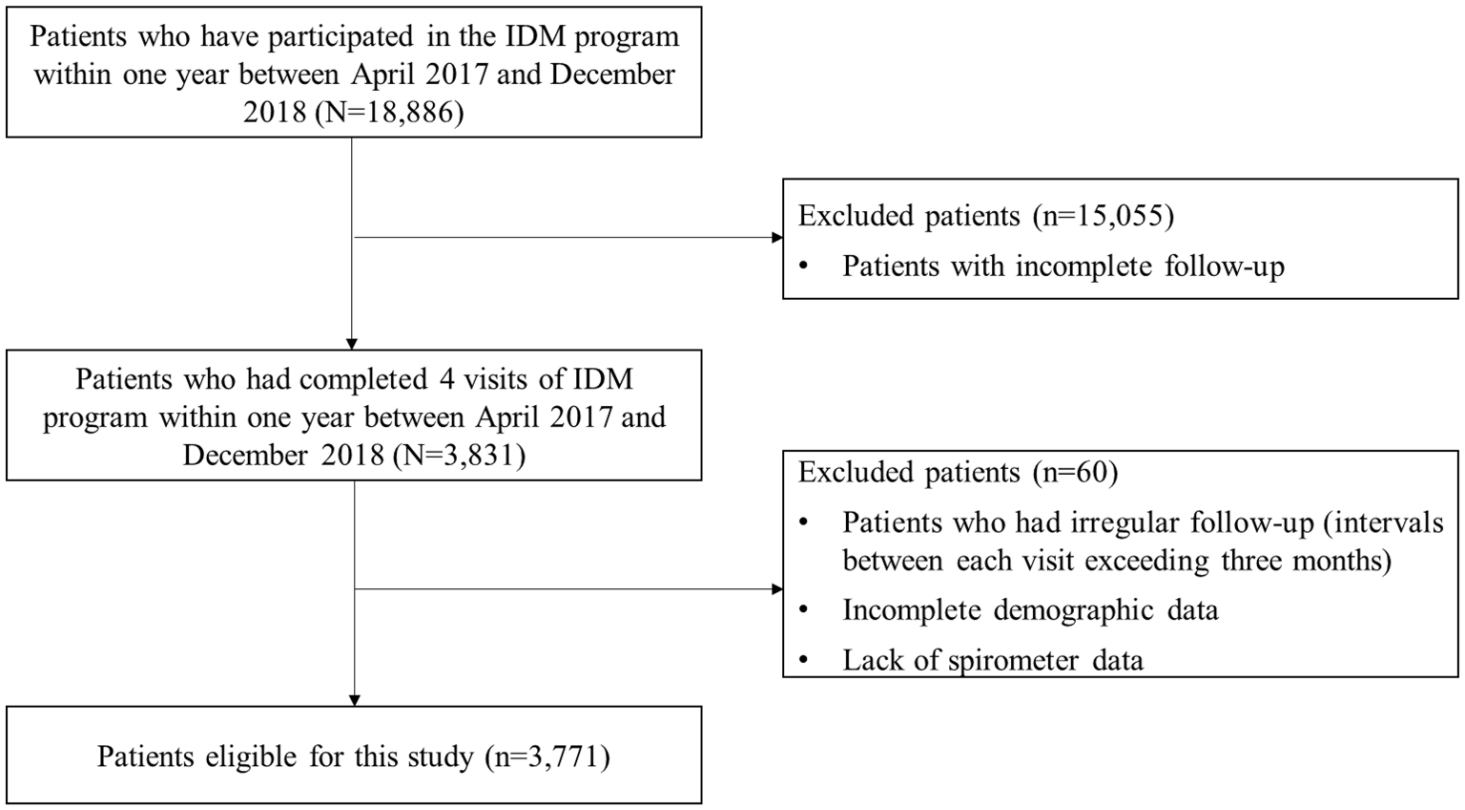
Study flowchart

Patients who had completed 4 visits of IDM program within 1 year between April 2017 and December 2018 were considered eligible for participation, yielding a total of 3831 participants in this retrospective study. COPD was defined as a confirmed COPD diagnosis (ICD-10 code J41-J44) and their diagnosis was confirmed using a spirometer (postbronchodilator FEV1/FVC < 70%) within a 90 day period before enrolled in COPD IDM program. We excluded patients who had irregular follow-up (intervals between each visit exceeding 3 months), or had incomplete demographic data, or lack of spirometer data. The remaining 3771 patients had regular follow-up visit every 3 months for 1 year were selected for further analysis. A flowchart illustrating subject selection for this study is shown in Fig. 1.

### Outcomes and relevant variables

The CAT score is an instrument to assess and quantify health-related quality of life and symptom burden in COPD patients. We used CAT MCID as the primary outcome to determine the association between IDM intervention duration and COPD-specific health status improvement (the MCID of the CAT is defined as a 2-point drop in the CAT score from baseline to follow-up) [11]. The secondary outcome was to investigate the incidence of COPD exacerbation events, including COPD-related ED visits or COPD-related hospitalization. Potential relevant confounding variables included demographics (age, gender, body mass index (BMI), smoking status, hospital accreditation level, NHI branch) and baseline clinical characteristics (post-bronchodilator spirometry test results, charlson comorbidity index score, airflow limitation severity, mMRC, CAT score, acute exacerbation history, GOLD risk group, wheezing, treatment status, inhaler therapy (short-acting bronchodilator (SABD; including SABA (short acting β2 agonist), SAMA (short-acting muscarinic antagonists), and combination of SAMA and SABA)); mono-therapy (LABA (long-acting β2 agonist) alone, LAMA (long-acting muscarinic antagonists) alone, ICS (inhaled corticosteroids) alone); dual-therapy (LABA/ICS or LABA/LAMA); triple-therapy (LABA/LAMA/ICS), and the switch in COPD medication). The severity of airflow limitation and the GOLD risk group are defined according to the 2017 Global Initiative for Chronic Obstructive Lung Disease (GOLD). Airflow Limitation Severity is categorized as: Mild: FEV_1_ ≥ 80% predicted; Moderate: FEV_1_ ≥ 50% predicted but < 80% predicted; Severe: FEV_1_ ≥ 30% predicted but < 50% predicted; Very severe: FEV_1_ < 30% predicted. Charlson comorbidity index score was used to quantify the severity of baseline comorbidity [12]. Baseline treatment status as defined by patient who with or without prescription bronchodilator before COPD IDM program enrollment.

### Statistical analysis

Descriptive data are reported as means ± SD or percentages as appropriate. Comparisons between groups for descriptive summaries were using chi-square tests for categorical variables and independent sample t test or Mann–Whitney U test, as appropriate, for continuous variables. Least-squares means (LSMeans) were used to assess change from baseline in CAT score. The Youden index were used to determine the cutoff values of COPD IDM intervention duration for improving the CAT score. Logistic regression analysis was used to examine the association between IDM intervention duration and improvement in CAT score that achieved MCID thresholds, and the factors associated with CAT MCID improvement. Cumulative incidence curve and Cox proportional hazards models was used to estimate exacerbations COPD exacerbation events, and results were reported as crude and adjusted hazard ratios. The data management, analysis, and visualization were conducted using SAS version 9.4 (SAS Institute Inc., Cary, NC, USA) or R software (version 4.1.0; The Comprehensive R Archive Network: http://cran.r-project.org). A two-tailed P value of 0.05 was considered statistically significant in all analyses.

## Result

### Patient characteristics at baseline

We observed a cohort of 3771 patients with COPD who had regularly completed 4 visits of IDM program within 1 year. Overall, the mean age was 71.47 ± 9.7 years, and 91.51% of patients were men, 27.5% of patients were current smoker, 42.7% of patients had CAT score of ≥ 10 at baseline and 45.48% of patients were classified as GOLD B. The mean CCI, FEV_1_, FEV_1_% predicted, FEV_1_/FVC ratio, mMRC, and CAT at baseline were 2.26 ± 1.53, 1.46 ± 0.56 L, 63.37 ± 22.08%, 56.67 ± 11.48%, 1.53 ± 0.91, and 10.49 ± 6.47, respectively. **(**Table 1**)**.

**Table 1:** Demographic and clinical characteristics

	N = 3771
Age, mean (SD)	71.47 ± 9.7
Age category (year), N (%)	
< 50	57 (1.5)
51–60	362 (9.6)
61–70	1173 (31.2)
71–80	1343 (35.7)
> 80	836 (22.2)
Gender, n (%)	
Female	320 (8.4)
Male	3451 (91.5)
Charlson comorbidities index, mean (SD)	2.26 ± 1.53
CCI category, n (%)	
0–1	94 (2.4)
2	2322 (61.5)
≥ 3	1355 (36)
BMI (kg/m^2^), mean (SD)	24.93 ± 46.55
BMI category (kg/m^2^), n (%)	
<18.5	276 (7.2)
18.5 ≤ BMI < 24	1768 (46.8)
≥ 24	1727 (45.9)
Smoking status, n (%)	
Never smoker	504 (13.5)
Former smoker	2240 (59.4)
Current smoker	1027 (27.3)
Accreditation level, n (%)	
Medical center	1255 (33.3)
Regional hospital	1800 (47.7)
District hospital	562 (15)
Clinics	154 (4.2)
Branch, n (%)	
Taipei	1182 (31.2)
Northern	457 (12)
Central	944 (24.9)
Southern	682 (18)
Kao-Ping	437 (11.7)
Eastern	69 (1.8)
Baseline FEV1 (L), mean (SD)	1.46 ± 0.56
Baseline FEV% (% of predicted value), mean (SD)	63.37 ± 22.08
Baseline FEV1/FVC (%), mean (SD)	56.67 ± 11.48
Baseline airflow limitation, n (%)	
≥ 80% predicted	841 (22.2)
50–79% predicted	1862 (49.5)
30–49% predicted	888 (23.4)
< 30% predicted	180 (4.8)
Baseline mMRC, mean (SD)	1.53 ± 0.91
Baseline mMRC category, n (%)	
0	469 (12.3)
1	1441 (38.1)
2	1313 (34.8)
3	505 (13.5)
4	43 (1.2)
Baseline CAT, mean (SD)	10.49 ± 6.47
Baseline CAT category, n (%)	
0–10	2162 (57.3)
11–20	1295 (34.2)
21–30	296 (7.8)
31–40	18 (0.6)
Acute exacerbation in previous 1 year, n (%)	
< 2	3239 (85.8)
≥ 2	532 (14.1)
Baseline GOLD risk group, n (%)	
A	1136 (30)
B	1715 (45.6)
C	314 (8.4)
D	606 (16.2)
Wheezing at baseline, n (%)	
No	3089 (81.9)
Yes	682 (18)
Naïve-treatment patients at baseline, n (%)	
No	3615 (96)
Yes	156 (4.2)
Inhaler therapy at baseline, n (%)	
Without inhaler prescription	22 (0.6)
SABD	43 (1.1)
Mono therapy	841 (22.3)
Dual therapy	2006 (53.2)
Triple therapy	859 (22.8)
Switch in COPD medication over the course of the program, n (%)	
No	2525 (67.0)
Yes	1246 (33.0)

Data are presented as mean ± SD or number (%)

*BMI* Body Mass Index, *CAT* The COPD assessment test, *CCI* Charlson comorbidities index, *FEV1* forced expiratory volume in 1 s, *FVC* forced vital capacity, *GOLD* global obstructive lung disease, *mMRC* Modified Medical Research Council Dyspnoea Scale, *SABD* short-acting bronchodilator (including short acting β2 agonist (SABA) or short-acting muscarinic antagonists (SAMA), and combination of SAMA and SABA), Mono-therapy (long-acting β2 agonist (LABA) or long-acting muscarinic antagonists (LAMA) or inhaled corticosteroids (ICS)), Dual-therapy (LABA/LAMA or LABA/ICS), Triple therapy (LABA/LAMA/ICS)

### The association between COPD IDM program duration and CAT score improvement

**Fig. 2:**
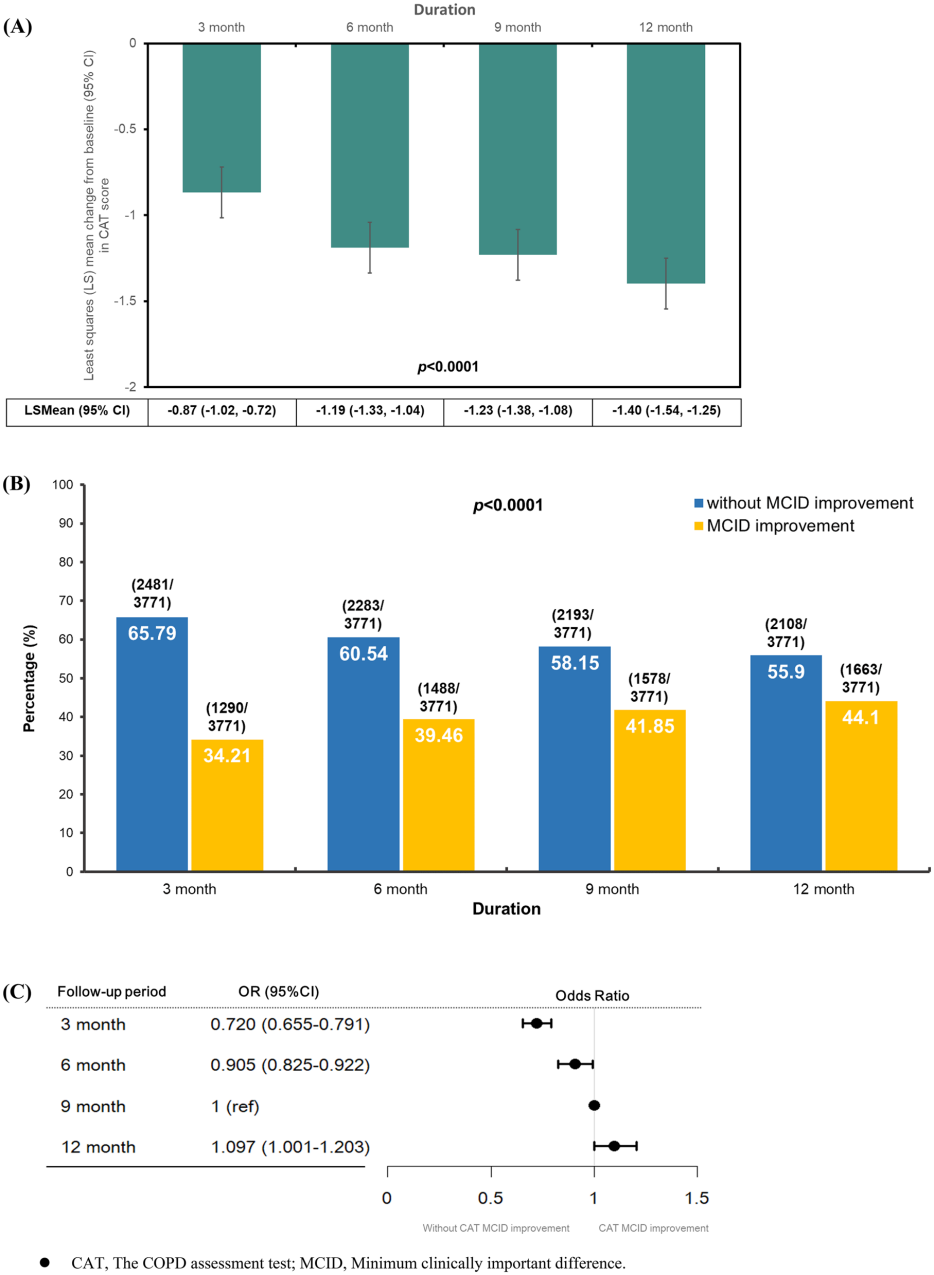
Change in CAT score from baseline to each timepoint (**A**) Least squares (LS) mean change from baseline (95% CI) in CAT score from baseline to each visit; (**B**) Proportion of patients with and without achieving MCID improvement in CAT score from baseline to each visit; (**C**) Odds ratio of patients with MCID improvement in CAT score from baseline to each visit. *CAT* The COPD assessment test; *MCID* Minimum clinically important difference

Changes in mean total CAT score for patients shown in Fig. 2A. The least squares mean change in CAT score from baseline visit were − 0.87 (95% CI − 1.02 to − 0.72), − 1.19 (95% CI − 1.33 to − 1.04), − 1.23 (95% CI − 1.38 to − 1.08), and − 1.40 (95% CI − 1.54 to − 1.25) points at 3-, 6-, 9- and 12 month follow-up (p < 0.0001 for all visits), respectively. Although statistically significant improvements in the LS mean change from baseline in CAT score were observed at all time points, the clinically meaningful improvements were not achieved (CAT score decreased for at least 2 points). To further evaluate the impact of COPD IDM program duration on clinically meaningful improvement in CAT score. We use CAT MCID as an indicator to measure the change in the proportion of patients who are achieving clinically meaningful improvement in CAT score at all time points. Figure 2B demonstrates that 34.21% of patients achieved MCID improvement in CAT scores at 3 months, followed by 39.46%, 41.85%, and 44.10% at 6-, 9 months, and 12 month follow-up, respectively, with a significant increase (p < 0.0001). Using the Youden index, we found that the cut-off value for COPD IDM program duration to achieve CAT MCID improvement was at 9 months. Logistic regression demonstrated that statistically significantly lower likelihood of achieving MCID improvement in CAT were observed at 3- and 6- month compared to 9 month (at 3 month: OR: 0.720, 95% CI 0.655–0.791; at 6 month: OR: 0.905, 95% CI 0.825–0.922). And only a modest increase likelihood of achieving MCID improvement in CAT at 12 month (OR: 1.097, 95% CI 1.001–1.203) compared with 9 month follow-up (Fig. 2C). Furthermore, our analysis revealed no significant interaction effect between different pharmacotherapy and intervention duration (interaction, p = 0.7334). This result indicated that stratified pharmacotherapy does not impact the improvement of CAT MCID over the course of the intervention.

### Factors associated with the patients who are achieving CAT MCID improvement

**Fig. 3:**
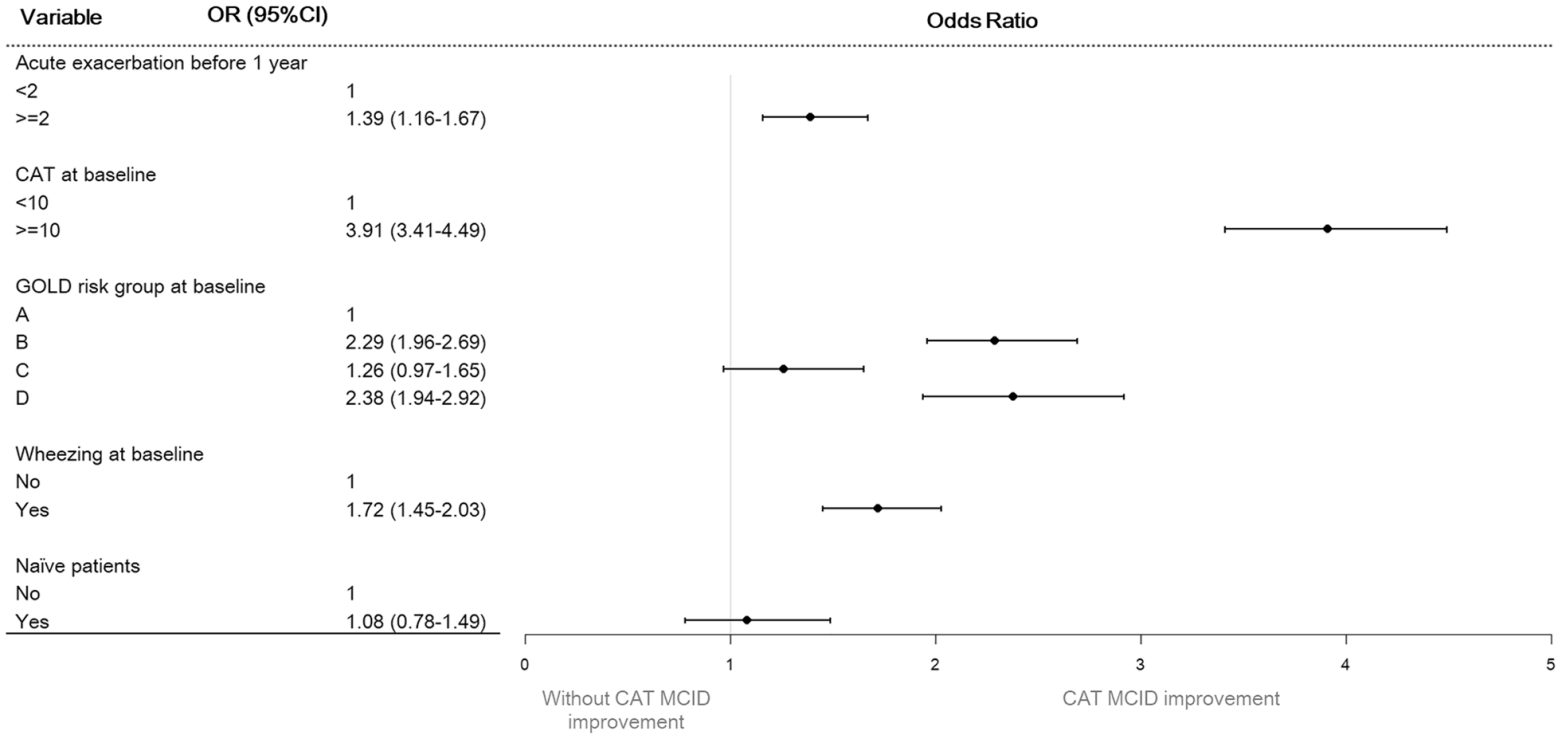
Factor associated with patients who achieve the CAT MCID improvement

Figure 3 showed the associations between factors and CAT MCID improvement. Logistic regression demonstrated that achievement of MCID improvement in CAT score were associated with patients who have had two or more COPD exacerbations (OR: 1.39, 95% CI 1.16–1.67), a baseline CAT score greater than 10 points (OR: 3.91, 95% CI 3.41–4.49), wheezing at baseline (OR: 1.72, 95% CI 1.45–2.03), or who are classified as GOLD B (OR: 2.29, 95% CI 1.96–2.69) or GOLD D (OR: 2.38, 95% CI 1.94–2.92) at baseline. The results indicate that CAT MCID improvement was most associated with baseline CAT scores, followed by frequent exacerbation in previous year (> 2 episodes/year), GOLD B or D, and wheezing at baseline.

### Changes in mean total CAT score for patients with CAT score < 10 points or CAT score ≥ 10 points

**Fig. 4:**
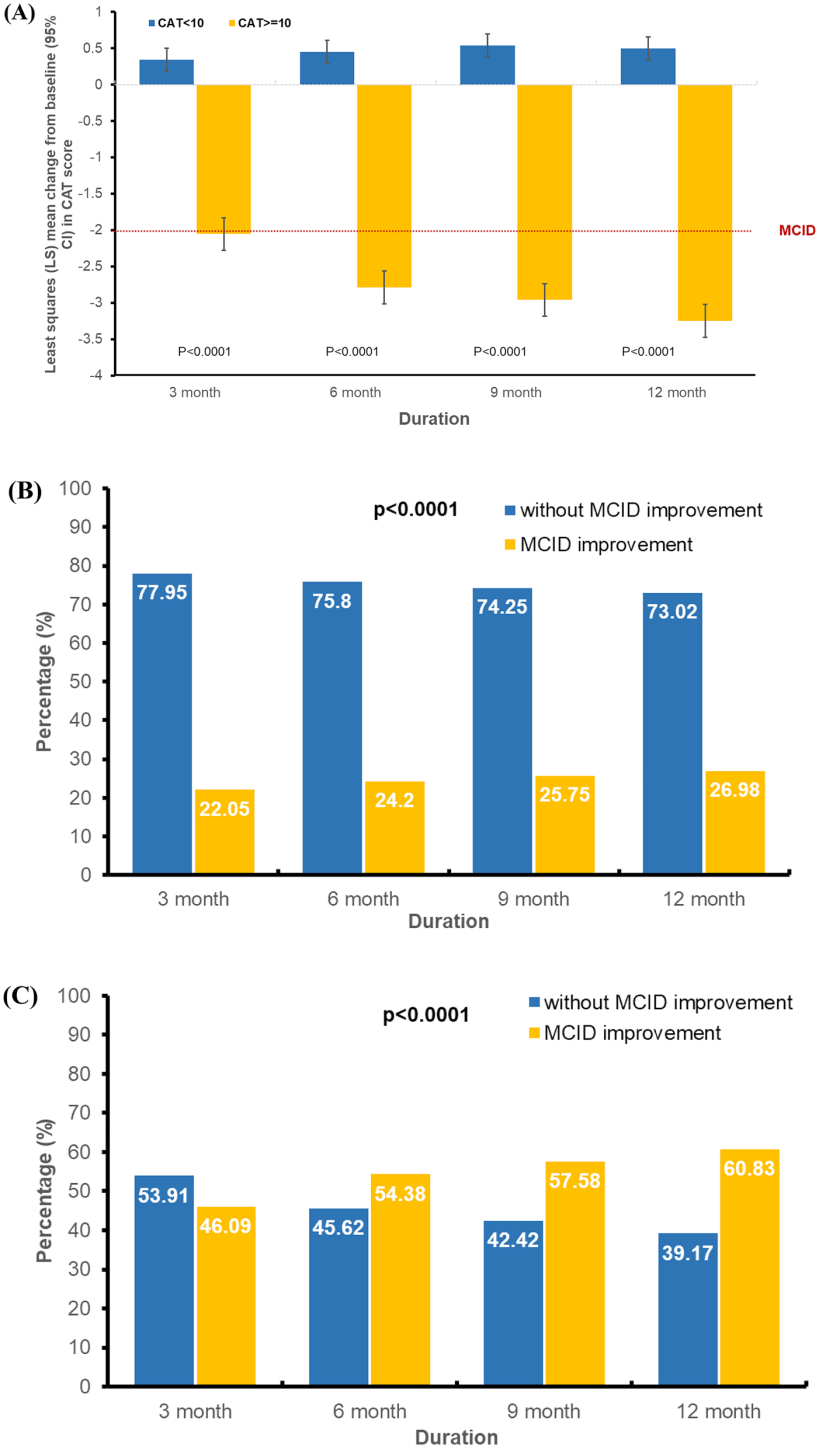

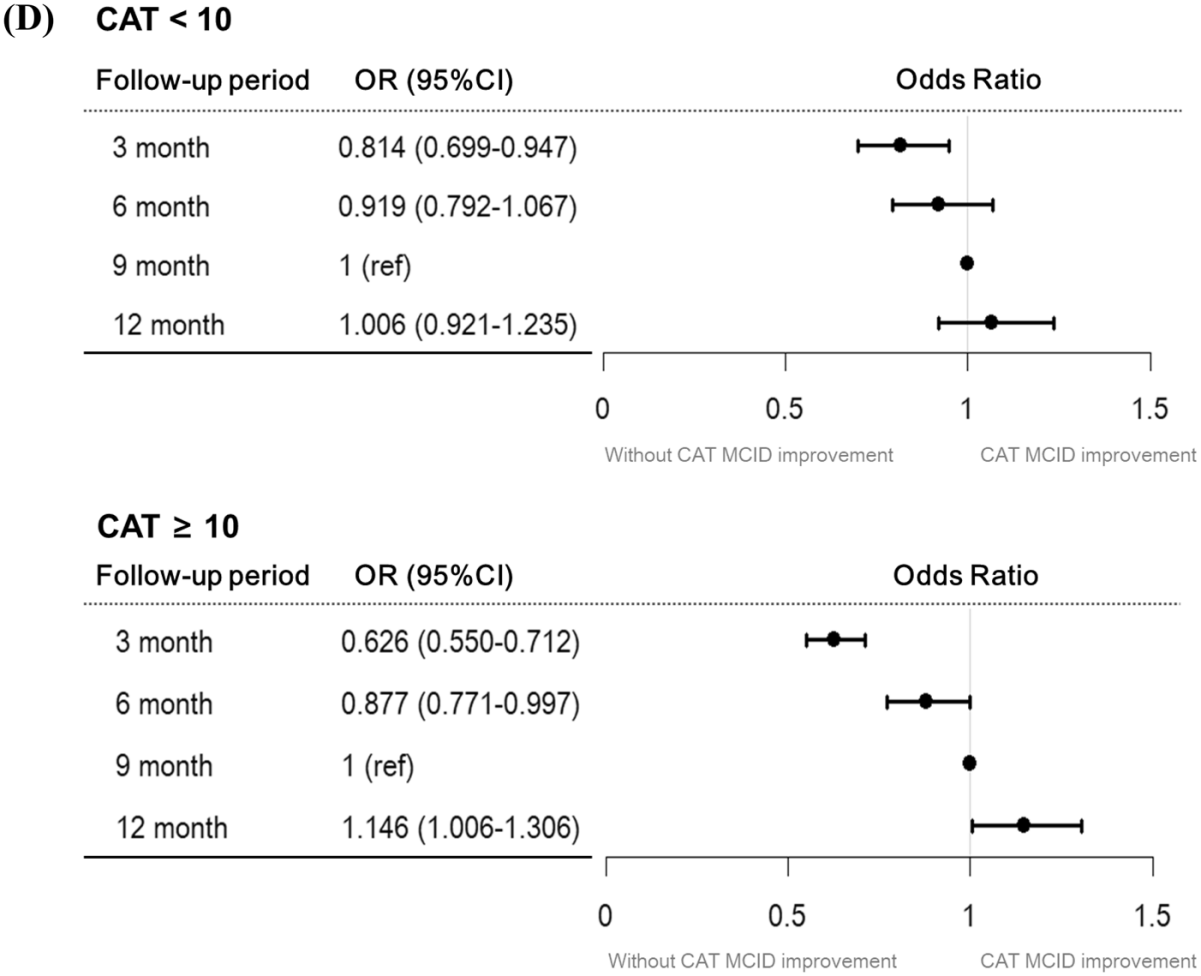
Change in CAT score from baseline to each visit for subgroups of patients with baseline CAT score < 10 points or CAT score ≥ 10 points. **A** Least squares (LS) mean change from baseline (95% CI) in CAT score from baseline to each visit; Proportion of patients with and without achieving MCID improvement in CAT score from baseline to each visit in (**B**) patients with baseline CAT score < 10 points and (**C**) patients with baseline CAT score ≥ 10 points; Odds ratio of patients with MCID improvement in CAT score from baseline to each visit. **D** patients with baseline CAT score < 10 points and patients with baseline CAT score ≥ 10 points

Changes in mean total CAT score for groups defined by total CAT score at baseline are shown in Fig. 4. In the CAT < 10 group, the least squares mean change in CAT score from baseline were not achieve CAT MCID improvement during study period. While, in the CAT ≥ 10 group, the mean change from baseline in total CAT score was − 2.05 (95% CI − 2.28 to − 1.83) at 3 months, continuously improving to − 2.789 (95% CI − 3.01 to − 2.57), − 2.96 (95% CI − 3.19 to − 2.74), and − 3.25 (95% CI − 3.47, − 3.02) at 6 month, 9 month, and 12 months (Fig. 4A). Figure 4B demonstrates, in CAT < 10 group, that proportion of patients who achieve CAT MCID improvement were significantly fewer than those who do not. While, in CAT ≥ 10 group, the proportion of patients achieved CAT MCID improvement was 46.09% at 3 months, continuously increasing to 54.38%, 57.58% and 60.83% at 6-, 9- and 12 month (Fig. 4C). The result indicated that a significantly statistical and clinical improvement of CAT score were observed in baseline CAT ≥ 10 group than baseline CAT < 10 group at all time point. Consistent with overall cohort, the cut-off value for COPD IDM program duration to achieve CAT MCID improvement was also at 9 months. In CAT ≥ 10 group, statistically significantly lower likelihood of achieving MCID improvement in CAT were observed at 3- and 6 month compared to 9 month (at 3 month: OR: 0.626, 95% CI 0.550–0.712; at 6 month: 0.877, 95% CI 0.771–0.997). And a modest increase likelihood of achieving MCID improvement in CAT at 12 month (OR: 1.146, 95% CI 1.006–1.306 at 12 month) compared with 9 month follow-up (Fig. 4D).

### Cumulative incidence and hazard ratios of COPD exacerbation events

**Fig. 5:**
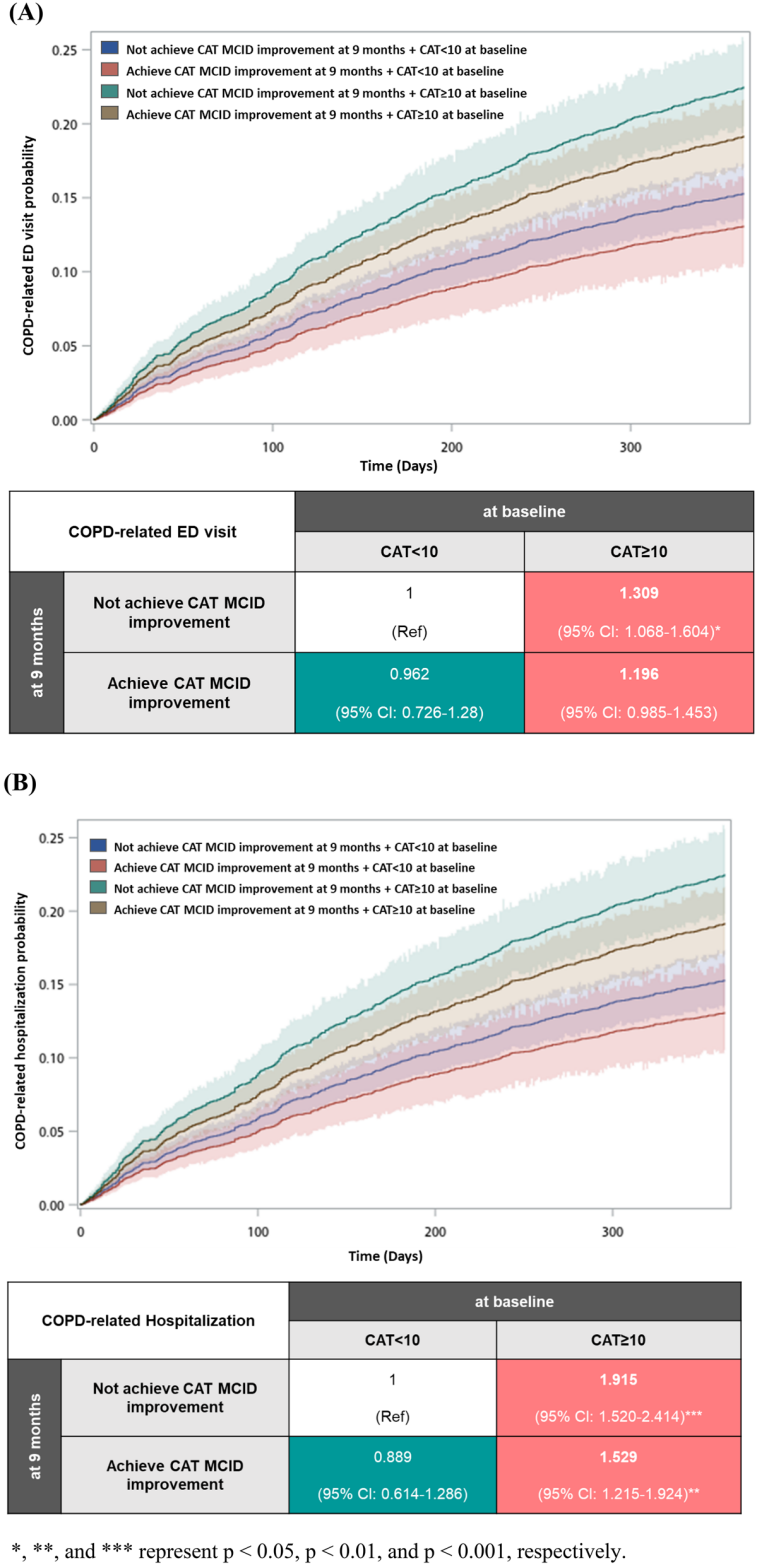
Cumulative incidence and hazard ratios of (**A**) COPD-related ER visits and (**B**) COPD-related hospitalizations within 1 year. *, **, and *** represent p < 0.05, p < 0.01, and p < 0.001, respectively

Cumulative incidence curves for estimating the further risk of COPD exacerbation events (COPD-related ED visit and COPD-related hospitalization) within the next year for subgroups defined by total CAT score at baseline and CAT MCID improvement shown in Fig. 5. Significant differences were found between the four study groups (p < 0.0001, using Gray’s test). Patient with CAT ≥ 10 at baseline was associated with a significantly increased incidence of subsequent COPD exacerbation events. Further, patients with CAT MCID improvement had a significantly lower incidence of COPD exacerbations than those without. In CAT ≥ 10 subgroup, patients achieved CAT MCID improvement had lower risk of COPD exacerbation (COPD-related ED visit: aHR: 1.196, 95% CI 0.985–1.453, p = 0.0713; COPD-related hospitalization: aHR: 1.529, 95% CI 1.215–1.924, p = 0.0003) than those without (COPD-related ED visit: aHR: 1.309, 95% CI 1.068–1.604, p = 0.0095; COPD-related hospitalization: aHR: 1.915, 95% CI 1.520–2.414, p < 0.0001), which is highest risk of COPD exacerbation in four subgroup.

### Sensitivity analysis

We further included all patients who have participated in the program at least once for sensitivity analysis. The results demonstrated that statistically significantly lower likelihood of achieving MCID improvement in CAT were observed in patient at 3- and 6 month compared to 9 month (at 3 month: OR: 0.595, 95% CI 0.516–0.685; at 6 month: OR: 0.784, 95% CI 0.678–0.907). And only a modest increase likelihood of achieving MCID improvement in CAT at 12 month (OR: 1.098, 95% CI 0.968–1.247) compared with 9 month follow-up (Additional file 1: Fig S1). These results are consistent with the main results in patients who completed a 1 year follow-up.

## Discussion

Our study is the first to demonstrates the association between the intervention duration of COPD IDM program and COPD-related outcomes in real-world setting. Our finding indicates that likelihood of achieving CAT MCID improvement is lower at 3- and 6 month compared to 9 month and a modest increase likelihood of improvement at 12- month compared with 9 month. CAT MCID improvement was most associated with baseline CAT scores. From 3 to 12 month follow-up results showed continued improvement over time in COPD-specific health status, particularly in patients with baseline CAT score of ≥ 10. In addition, patients with CAT MCID improvement had lower risk of subsequent COPD exacerbation.

The meta-analysis summarized the literatures analyzing 52 trials involving 21,086 participants from 19 countries in a variety of health care settings with the follow-up periods ranged between 3 and 48 months, concluding that IDM probably improves health-related QoL in medium-term follow-up (> 6 to 15 months) [3]. In our study, we found that IDM interventions continuously improves COPD-specific QoL over 1 year and patients were likely to achieve MCID improvement in CAT score after 9 month- follow-up. Compared to previous meta-analysis, our real-world study provides direct evidence that the amount of time between baseline and follow-up influenced the change of QoL at follow-up, which supports that IDM intervention duration longer than 6 month significantly ameliorate COPD-specific QoL. This study measured QoL using the CAT score since it is a concise and validated questionnaire that can easily be implemented instead of the St. George’s Respiratory Questionnaire (SGRQ) used in Poots' meta-analysis. Although it is difficult to directly compare the magnitude of the effect between our study and those in the Poots’ meta-analysis study due to the use of different QoL tools. The St. George’s Respiratory Questionnaire (SGRQ) and the CAT score perform similarly have been validated by previous studies [13, 14]. Conservatively, the improvements in QoL measured in our study are consistent with those reported in previous research on COPD IDM intervention.

In Poots meta-analysis study, the mean improvement in QoL measured by the SGRQ was 3.89 which do not achieve MCID for the SGRQ at medium-term follow-up. Similar to our study, the mean improvement in total CAT score also do not achieve CAT MCID threshold (≥ 2 points reduction) during 1 year study period. Previously, Ferrone et al. study evaluated the IDM intervention in a high risk, frequent exacerbation population with a poor baseline QoL (mean CAT score of all subjects was 21). Result from the study demonstrated that QoL improved in the IDM cohort with a CAT score of 22.6 at baseline and 14.8 at 12 months, whereas The CAT score increased from 19.3 to 22.0 in the usual care arm [15]. The authors confirmed that an IDM intervention substantially improved QoL in a high-risk primary care population. Therefore, we suggest that IDM intervention may have more beneficial effect on patients with poor conditions at baseline than those are not. Patients with CAT score of ≥ 10 were indicating medium-to-very high impact of COPD on the patient’s life has been reported [16, 17]. Our result found that patients with CAT score ≥ 10 were more likely to achieve CAT MCID improvement. Moreover, in CAT score ≥ 10 group, the mean changed from baseline in CAT score at all 4 time points with 1 year were greater than − 2 point (ranged from − 2.05 to − 3.25 point), which achieved MCID threshold. These findings support that patients with poor baseline conditions, particularly CAT score ≥ 10 at baseline, are the potential beneficiary group of COPD IDM program by improving COPD-specific QoL for reducing the impact of COPD on the patient’s life.

Besides CAT score at baseline, the other factors associated with MCID improvement in CAT score included frequent exacerbation (2 episodes/year), wheezing, and GOLD B and D. In previous study, the integrated care program had a significant beneficial impact on health status in patient with frequent exacerbator, with a reduction in CAT score from 19 to 15 after 1 year of follow-up [18]. In addition, patients enrolled in the IDM program classified as GOLD B and D at baseline had more improvement in CAT scores than GOLD A and C after 1 year follow-up [19]. According to these evidence, at baseline condition, patients with CAT score ≥ 10, frequent exacerbation, or GOLD B and D are the potential candidate who may had more benefit from IDM intervention.

Patients with CAT score of ≥ 10 at baseline had higher exacerbation risk compared to those with CAT < 10 at baseline has been identified in previous study [20]. Similar to our finding, patients with CAT score of ≥ 10 (both patient with or without CAT MCID improvement) had higher risk of COPD exacerbation event, including ED visit and hospitalization. However, patients (with CAT score of < 10 or ≥ 10) who achieve CAT MCID improvement significantly had lower risk of subsequent COPD exacerbation event compared to those did not. In previous study, the group with improved CAT score (patients who exhibited a decrease of 2 points or more) had significantly fewer moderate-to-severe exacerbations than the those without improvement during the short-term (approximately 6 months) follow-up after bronchodilator therapy [21], which is similar with our result. Moreover, patients with stable or improved health status during 1 year follow-up had a lower likelihood of exacerbation also has been reported [22]. These findings support that patients who participated in IDM program with MCID improvement in CAT score are likely to reduce the risk of subsequent COPD exacerbation events. In addition, the change in CAT score provides a simple estimate of exacerbation risk during IDM intervention, providing useful information for maintaining patient stability.

There are some limitations to this study. First, the study findings may not be generalizable to other countries or populations due to the unique health care system and COPD P4P model in Taiwan. Second, since adherence evaluations are based on claims data, we assumed that filled prescriptions are proxies for medication adherence. It must be noted, however, that a filled prescription is not proof that the patient took it. Due to our assumption that every filled prescription was fully taken, our estimated medication adherence rates overestimate real medication adherence. Third, only 1 year follow-up was included in our study, so assessing the P4P program’s effect on mortality was difficult. Forth, the implementation of pulmonary rehabilitation program depends on the physicians’ judgement and patient's clinical condition. Moreover, record of home-based pulmonary rehabilitation is unavailable in NHIRD. Therefore, rate of participating pulmonary rehabilitation may underestimate.

## Conclusion

This is the first real-world study indicating the association between intervention duration of COPD IDM program and COPD-related outcomes. There are continued improvement over time in COPD-specific health status from 3 to 12 month follow-up, particularly in patients with baseline CAT score of ≥ 10. In addition, a reduction of the risk of subsequent COPD exacerbations were observed in patients with CAT MCID improvement.

## Supplementary Information

**Additional file 1: Fig S1. **Sensitivity analysis: odds ratio of patients with MCID improvement in CAT score from baseline. **Table S1. **Arithmetic mean change from baseline in CAT score to each visit for overall cohort, subgroups of patients with baseline CAT score <10 points or CAT score ≥10 points.

## Data Availability

Clinical research data from Applied Health Research Data Integration Service from National Health Insurance Administration supported the findings of this study. Restrictions apply to the availability of these data and they are therefore not publicly available.
