# Supplementary material for: The impact of the duration of the integrated disease management program on COPD-related outcomes
Source: Eur J Med Res. 2023 May 23;28:178. doi: 10.1186/s40001-023-01136-0 (PMC10204265; doi:10.1186/s40001-023-01136-0)
Supplement: Supplementary file 1 — Additional file 1: Fig S1. Sensitivity analysis: odds ratio of patients with MCID improvement in CAT score from baseline. Table S1. Arithmetic mean change from baseline in CAT score to each visit for overall cohort, subgroups of patients with baseline CAT score <10 points or CAT score ≥10 points. [file 40001_2023_1136_MOESM1_ESM.docx]

**Figure S1.** Sensitivity analysis: odds ratio of patients with MCID improvement in CAT score from baseline


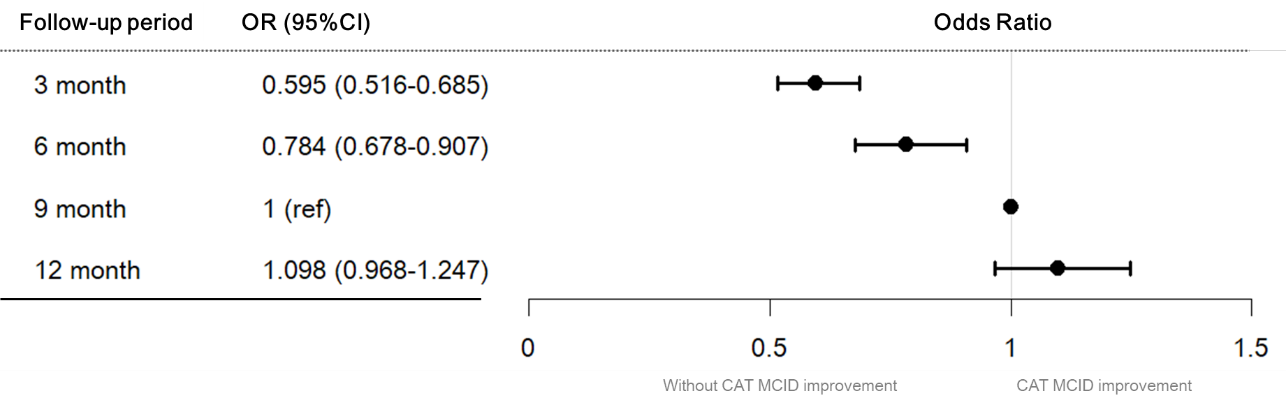


**Table S1.** Arithmetic mean change from baseline (±SD) in CAT score to each visit for overall cohort, subgroups of patients with baseline CAT score <10 points or CAT score≥10 points

| **Mean change from baseline (±SD) in CAT score** | **Overall** | **baseline CAT scores <10** | **baseline CAT scores ≥ 10** |
| --- | --- | --- | --- |
| 3 month | -0.87±4.03 | 0.35±3.24 | -2.05±4.39 |
| 6 month | -1.19±4.49 | 0.45±3.42 | -2.79±4.82 |
| 9 month | -1.23±4.86 | 0.54±3.61 | -2.96±5.28 |
| 12 month | -1.40±5.03 | 0.50±3.69 | -3.25±5.46 |
